# The Nkateko health service trial to improve hypertension management in rural South Africa: study protocol for a randomised controlled trial

**DOI:** 10.1186/1745-6215-15-435

**Published:** 2014-11-07

**Authors:** Margaret Thorogood, Jane Goudge, Melanie Bertram, Tobias Chirwa, Sandra Eldridge, Francesc Xavier Gómez-Olivé, Felix Limbani, Eustasius Musenge, Nokuzola Myakayaka, Stephen Tollman, Rhian Twine

**Affiliations:** Division of Health Sciences, Warwick Medical School, University of Warwick, Gibett Hill Road, Coventry, CV4 7AL UK; MRC/Wits Rural Public Health and Health Transitions Research Unit, School of Public Health, Faculty of Health Sciences, University of the Witwatersrand, Education Campus, 27 St Andrew’s Road, Parktown, 2193 Johannesburg, South Africa; Centre for Health Policy, School of Public Health, Faculty of Health Sciences, University of the Witwatersrand, Education Campus, 27 St Andrew’s Road, Parktown, 2193 Johannesburg, South Africa; World Health Organisation, Avenue Appia 20, 1211 Geneva 27, Switzerland; Division of Epidemiology and Biostatistics, School of Public Health, Faculty of Health Sciences, University of the Witwatersrand, Education Campus, 27 St Andrew’s Road, Parktown, 2193 Johannesburg, South Africa; Queen Mary University of London, Mile End Rd, London, E1 4NS UK; Umeå Centre for Global Health Research, Division of Epidemiology and Global Health, Department of Public Health and Clinical Medicine, Umeå University, Förvaltningshuset, Universitetsorget 16, 901 87 Umeå, Sweden; International Network for the Demographic Evaluation of Populations and Their Health (INDEPTH), 38 & 40 Mensah Wood Street, East Legon, Accra, Ghana

## Abstract

**Background:**

South Africa has a high and rising prevalence of hypertension. Many affected individuals are not using medication, and few have controlled blood pressure. Until recently, primary care clinics focused on maternal and child health and management of acute conditions, but new government initiatives have shifted the focus to chronic diseases, including HIV/AIDS and hypertension.

**Methods/Design:**

The Nkateko trial will test the effectiveness of clinic-based lay health workers (LHWs) in supporting hypertension management. It is a pragmatic, cluster randomised controlled trial based in the Agincourt subdistrict of northeast South Africa, and it is underpinned by long-term health and demographic surveillance. Eight primary care facilities, with their catchment communities, are randomised to usual care or the addition of LHWs focused on chronic care. All clinics (intervention and control) will be provided with a clerk to collect information on clinic attendees and will match them to preexisting surveillance records. Intervention clinics will have LHWs working alongside nursing staff and focusing on health care for people with chronic conditions, particularly hypertension. The LHWs will be supported by an implementation manager, who will work with clinic staff to develop the most effective role for the LHWs. Control clinics will continue to provide usual care. The primary outcome will be the change between two population surveys conducted before and after the intervention in the proportion of the population with uncontrolled hypertension and a risk profile indicating at least moderate risk of cardiovascular disease. A process evaluation will be based on a realist approach using patient exit interviews, clinic observations and interviews with health professionals, LHWs and patients to document the intervention and its implementation.

**Discussion:**

There are challenges in the design of this trial. Assessing change through population surveys may reduce measurable effects; however, we feel this is appropriate because we aim to attract those who currently do not use clinics, and we hope to improve care for clinic users. Clinics were randomised at an open meeting because we were concerned that a remote process of randomisation would not be trusted by the community. We are constantly working to achieve an effective balance between the intervention and process evaluations.

**Trial registration:**

ISRCTN12128227 (registered 5 March 2014)

**Electronic supplementary material:**

The online version of this article (doi:10.1186/1745-6215-15-435) contains supplementary material, which is available to authorized users.

## Background

There is a complex epidemiological and demographic transition underway in sub-Saharan Africa. The HIV/AIDS epidemic, which has affected a large proportion of the population and caused a dramatic decrease in life expectancy, coexists with an ageing population and an increase in the prevalence of noncommunicable diseases. It is expected that the proportion of the population 60 years of age and older will increase almost threefold by 2025 compared to 1985 data [1]. At the same time, partly due to this demographic shift, South Africa has a high and rising prevalence of hypertension [2, 3]. Hypertension currently affects around 40% of the adult population in the Agincourt subdistrict, a rural area covered by the Agincourt Health and Demographic Surveillance System (HDSS) (unpublished data; Clark S, Gómez Olivé FX see also [4]. In low-resource rural settings, fewer than half of individuals affected by hypertension are aware that they have hypertension, and fewer than 10% achieve appropriate blood pressure levels [5]. There is marked variation in the management of hypertension and other chronic diseases, owing to poor functioning of primary care services, which have historically been focused on maternal and child health and the management of acute illness rather than chronic conditions. Adherence to medication is suboptimal; long-term patient retention is low; and little attention is paid to potential comorbidities [1].

South Africa also has the highest prevalence of HIV/AIDS in the world [6]. The country’s antiretroviral treatment programme is the largest worldwide, and it has seen recent notable increases in coverage [7]. Substantial knowledge has been generated on adherence support, tracing defaulters and enabling patient participation through treatment literacy and patient support groups [8]. This knowledge and experience may potentially be transferable to the management of hypertension, for which the same type of health care intervention may be equally effective in addressing the escalating burden. Treatment of HIV and tuberculosis relies heavily on lay health workers to support patient adherence. In South Africa, lay health workers have successfully (1) provided pre- and posttesting and adherence counselling, (2) assisted patients in navigating their engagement with health staff and (3) helped in tracing defaulters [9–12].

The development of this trial has been informed by the experience of the antiretroviral service and guided by the Wagner conceptual framework for chronic disease care, which emphasise the need for productive interactions between patients, providers and the broader health system [13]. Effective chronic disease care requires a reliable drug supply, longitudinal patient records to monitor care over time and adequately staffed clinics. It is also important that patients have sufficient self-efficacy to manage their illness with support from their social network. In an area such as the Agincourt subdistrict, where more than 90% of all adults have access to a mobile phone, text messaging could be used to improve medication adherence and health-related behaviour modification. There is evidence from a systematic review that text messaging was well accepted and showed early efficacy in most studies by improving medication adherence and health-related behaviour modification [14].

A recent national Department of Health initiative has sought to implement an integrated chronic disease management (ICDM) programme in 50 selected clinics in 3 provinces [15]. The aim of the ICDM programme is to strengthen chronic care by providing additional nurse training, equipment audit and replacement, improved drug supply, reorganised patient flow to allow separate management of chronic diseases and reduction of waiting times, advanced booking, and prepacked medication. The ICDM programme is already operational to a varying extent in the eight clinics in which this trial is being implemented.

Process evaluations are an important addition to randomised trials; they allow better understanding of the causal processes which may facilitate or obstruct change and help to inform the interpretation of outcomes [16, 17]. This is particularly important in a complex trial such as this one, where the interaction between the lay health workers, health care professionals, patients and the wider community is likely to be many-layered and where the interaction between the communities and the primary care clinics that serve them is poorly understood.

## Methods/Design

The full version of the study protocol is available at http://www.chp.ac.za/research/Nkateko/Pages/default.aspx.

### Research hypothesis and objectives

The hypothesis is that the introduction of lay health workers to assist nurses with the management of patients with chronic diseases in rural primary care clinics will improve population-level management of hypertension by improving diagnosis, retention in care and adherence to treatment by individuals with hypertension.

The following are our research objectives:

1. To compare the effectiveness of clinic-based lay health workers to ‘usual care’ in improving the management of hypertension (including access to care, adherence to treatment, and management) in rural South Africa
2. To conduct a realist evaluation to understand the patient, intervention, implementation, health care system and community barriers and facilitators that explain patient outcomes in the intervention and ‘usual care’ clinics
3. To contribute specific recommendations to strengthen policy and practice in similar rural settings of South Africa and southern Africa.

### Study design

**Figure 1:**
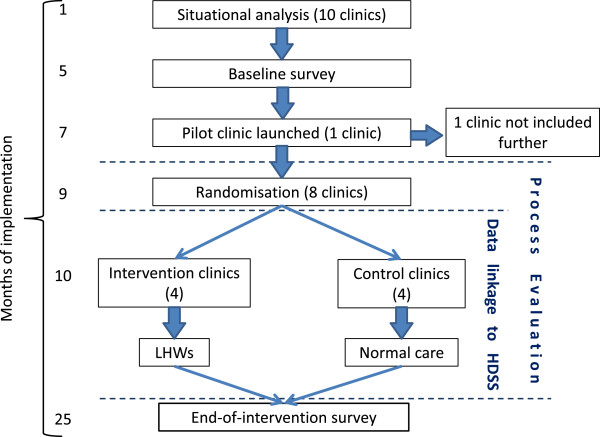
**Design of the Nkateko trial.** HDSS, Agincourt Health and Demographic Surveillance System; LHW, Lay health worker.

The Nkateko trial is a cluster randomised controlled trial comprising eight clusters each of which consists of a primary health care facility and the community it serves, including residents in a defined catchment area within the Agincourt subdistrict. Figure 1 shows a schematic representation of the trial.

In the trial, we are testing a health service intervention which involves placing lay health workers in four intervention clinics. The lay health workers will assist clinic nurses with the management of patients with chronic diseases. The outcome of the study will be evaluated by using two population-based, random sample, cross-sectional surveys, one at baseline and one at the conclusion of the intervention.

The study has been approved by the Committee for Research on Human Subjects (Medical), the University of the Witwatersrand (reference M130347, M130754, M130964), the Biomedical Research Ethics Committee at the University of Warwick (reference REGO-2013-062, REGO-2013-203, REGO-2013-562) and the Mpumalanga Province Research and Ethics Committee (dates of letters: 7 June 2013, 18 September 2013, 6 November 2013). This cluster randomised trial is a type A trial as defined in the 2002 Medical Research Council (MRC) document on cluster randomised trials [18]. The decision to consent to organisational change in a clinic cannot depend on the consent of users of the clinic. Consent has been obtained from the national, provincial and district authorities, and discussion of implementation within each clinic has taken place in partnership with the clinic staff. We have put notices in all clinics explaining that the clinic is being observed. Individual informed consent is obtained from each participant who is individually approached. This includes all clinic patients who allow their clinical data to be collected and linked to their existing demographic surveillance data, all participants in the two cross-sectional surveys, everyone (health service staff, research staff and community members) who engage in an interview or authorise a consultation to be observed, and all patients completing an exit questionnaire when they leave a clinic.

### Preintervention preparation

First steps in developing plans for the intervention were to carry out literature searches for similar interventions and to meet with local and provincial health service personnel. We then performed a situation analysis in all the local clinics. This involved interviews with clinic staff and collecting and analysing retrospective data on the appointments system and attendance of patients with chronic diseases. We also deployed local trained field workers to conduct nonparticipant observations over a period of 3 days in each clinic. We also held meetings with provincial and district stakeholders and workshops with staff in each clinic to feed back the results of our preparatory work to discuss how the interventions might proceed.

### Study site

The trial is based in the Agincourt subdistrict of Mpumalanga Province, South Africa. This rural area is a former Bantustan, with limited resources, high levels of unemployment and high levels of labour migration [19, 20]. In a population-based survey of 3,729 adults older than 15 years of age in 2010, the prevalence of hypertension was found to be 40% in women and 39% in men, and the prevalence of HIV was 27% in women and 19% in men (unpublished data). There are seven publically funded clinics and one health care centre at the site, plus two clinics just over the border. For this trial, one clinic from the border area, together with the population it serves (a population partly in the HDSS area), will be included as a pilot clinic. The other border clinic will not be considered further. The eight facilities within the site, and with their associated communities, will comprise the clusters. Until recently none of the health facilities kept individual longitudinal patient records. Moreover, individuals are free to use any clinic that is convenient, so, although each clinic has its own catchment area, it is not possible to define a ‘clinic user’ population for each clinic *a priori*.

Since 1992, the MRC/Wits Agincourt Research Unit has collected population data, and vital events (pregnancy outcome, death, migration) are updated yearly [19]. For the purpose of this study, the total population under surveillance is about 90,000 people (52,592 older than 18 years of age) who live in 15,500 households in 26 villages. These census data provide the sampling frame for the planned population surveys and also make it possible to trace patterns of clinic use by identifying clinic users at the facility levels and linking them to the population database. Moreover, the MRC/Wits Agincourt Research Unit provides an administrative research structure to support the trial.

### Intervention and control arms

All eight clinics will continue to implement the government’s ICDM strategy by providing separate care for patients with chronic diseases. In addition, as part of the trial, all clinics will have an attached data clerk responsible for collecting identifiers of all consenting attendees of the chronic disease clinic to allow their identification in the MRC/Wits Agincourt surveillance database. The linked data will enable us to understand patterns of clinic use which may not be geographically determined, as well as differential clinic use associated with sex, age and relative household wealth of clinic users. They will also allow us to monitor whether patterns of clinic use change over the 15 months of the intervention. Previous research [21] has confirmed the key identifiers for posterior links with the census are name, surname, age or date of birth, sex, village of residence, cell phone number, national ID number and name of another person living in the household. The system was piloted successfully in two clinics in 2013 in preparation for the Nkateko trial.

Intervention clinics will also be provided with two full-time lay health workers for 15 months to assist in the management of the patients with chronic diseases, particularly patients with hypertension. Building on experience with lay counsellors in antiretroviral therapy delivery [9], we expect that the lay health workers will provide adherence counselling; help to improve treatment literacy; use text messaging, telephone or personal contacts to remind patients of appointments [22]; and assist with filing of patient records and prepacking of medications. However, the exact roles of the lay health workers will be decided on a clinic-by-clinic basis in workshops run with the clinic staff by the implementation manager, with modification of noncore components to encourage local ownership. Core nonmodifiable components include appropriate staff selection, preservice and in-service training, and staff and programme evaluation. A local experienced nurse has been appointed as the implementation manager who will oversee the selection, introduction and functioning of the lay health workers. We believe this mirrors the type of structure that might be used by a provincial or district health department when introducing such an initiative. The implementation manager is responsible for the development of a functional relationship between the lay health workers and clinic staff, as well as the performance management and evaluation of the lay health workers.

### Study outcomes

We aim to improve the management of hypertension with the goal of achieving a reduction in the proportion of the population with uncontrolled hypertension and a risk profile indicating at least moderate risk of cardiovascular disease. To do this, we aim to both increase the number of people receiving active management of their hypertension and improve the management of blood pressure in those patients already in active management. Because we hope to reach people not currently using the clinics, we have chosen to measure the outcome of the trial at the population level with the use of two population surveys (before and after the intervention).

The primary outcome we will measure is the difference in the change in intervention and control clinics between the two surveys in the proportion of the population who have uncontrolled hypertension and a risk profile indicating at least moderate risk of cardiovascular disease. The definition of this risk profile is shown in Table 1. It reflects the concepts in the 2011 South African guidelines [23], which call for a focus on people at moderate or greater cardiovascular risk. Although the guidelines require clinical diagnoses, we are limited by what is possible in a population survey, so, for the purposes of this trial, we will define the cardiovascular risk factors and associated clinical conditions as outlined in Table 2.

**Table 1:** **Modified South African Guideline: stratification of cardiovascular risk in patients with hypertension** ^**a**^

	Presence of risk factors or other conditions			
Blood pressure (mmHg)	No risk factors	One or two risk factors	Three or more risk factors or diabetes	Associated clinical conditions
SBP 140 to 159 or DBP 90 to 99	Low added risk	Moderate added risk	High added risk	Very high added risk
SBP 160 to 179 or DBP 100 to 109	Moderate added risk	Moderate added risk	High added risk	Very high added risk
SBP 180+ or DBP 110+	High added risk	Very high added risk	Very high added risk	Very high added risk

^a^DBP, Diastolic blood pressure; SBP, Systolic blood pressure. Hypertension is defined as SBP >139 mmHg or DBP >89 mmHg.

**Table 2:** **Risk factors and associated clinical conditions used for definitions of ‘increased cardiovascular risk associated with hypertension’ in the Nkateko trial** ^**a**^

Risk factors	Associated clinical conditions
Sex/age: men >55 yr, women >65 yr	Self-reported coronary heart disease
Smoking at least every day	Self-reported heart failure
Dyslipidaemia TC >5 mmol/l	Self-reported stroke or TIA
Family history of CVD (sex/age): men <55 yr, women <65 yr	
Waist circumference: men >94 cm, women >80 cm	

^a^CVD, Cardiovascular disease; TIA, Transient ischaemic attack. TC. total cholesterol.

The secondary outcomes of the trial are shown in Table 3.

**Table 3:** **Secondary outcomes**

Source	Outcome
Before and after population surveys	Difference in change in proportion of the population with undiagnosed hypertension.
	Difference in change in the proportion of the population reporting they had had their blood pressure measured.
	Difference in change in the proportion of the population reporting they are using medication for hypertension.
	Difference in changes in the proportion of the population at different levels of blood pressure–related cardiovascular risk by age group and sex.
	Difference in change in the proportion of the population reporting they attended a clinic in the past year.
Clinic link with census records	Difference in proportion of population with diagnosed hypertension who are adherent to prescribed medication, defined by recorded collection of prescriptions.
	Difference in retention in care of people with diagnosed hypertension defined by the proportion of appointments kept during the study period.

### Power calculations, sample size and randomisation

In the absence of relevant data, we made the assumption that clinic use is equally distributed among the clinics. Each of the two population surveys will include around 4,000 participants from among a sample of 5,000, based on an estimated 80% response, and will give us approximately 500 people in each clinic (cluster). We adopted the use of the coefficient of variation as employed in similar study settings when a good intracluster variation is not available [24–26]. Data collected in Agincourt in 2010 provided a prevalence of moderate or greater cardiovascular risk of 36% and a coefficient of variation of 0.132 (error margin, 4.5% (0.132 ± 0.045)). Using these data, we calculated that we will have 88% power to detect a difference between an unchanged prevalence of risk of 36% in the control arm and a reduction to a prevalence of risk of 25% in the intervention arm. These calculations assume that the coefficient of variation will be similar in the two groups and that effects of the interventions are similar across clusters.

For each of the two population surveys, we will select a random sample of 5,000 people older than 18 years of age from the demographic surveillance database. The samples for the two surveys will be disproportionately stratified to ensure adequate representation of men and older people. This is necessary because (1) the population pyramid is heavily weighted toward younger people, and (2) there are fewer men than women amongst older adults due to labour migration [20] and the longer survival of women. Each sample will be independently selected, but we expect some overlap (estimated at around 4 to 500 individuals) between the two samples. All the individuals selected in both samples will be identifiable.

Randomisation of the eight clinics has been carried out at a public meeting attended by 38 members of the community, including clinic staff, lay members of clinic committees and members of a community advisory group. This was done to ensure that the community understands the purpose of the trial and that the clinic staff and wider community are confident that the randomisation was truly random. Eight pieces of paper with the names of the clinics were shown to attendees at the meeting, then put into sealed envelopes that were put in a box, which was shaken repeatedly before each envelope was chosen. The first clinic chosen was an intervention clinic, the second a control clinic and so forth.

### Baseline and end-of-intervention population surveys

All consenting participants in the surveys will have their blood pressure measured by trained field staff three times using an Omron automatic blood pressure machine (model M6W; Omron Healthcare, Lake Forest, IL, USA). The questionnaire takes around 30 minutes to administer, thus allowing both surveys to be completed within a 12-week period before the start of the intervention and at the end. Information will be collected regarding respondents’ use of primary care clinics in the past 12 months and their preferred clinic. Information on factors related to cardiovascular risk will be collected, and respondents will also be asked if they have had their blood pressure checked by a doctor or nurse in the past year, if they have ever been told they have hypertension and if they are using medication for hypertension (see Additional file 1 for a copy of the questionnaire).

### Data management and analysis

Quantitative data will be entered at the field site using double data entry. Personal identifiers will be encrypted once the data have been entered and cleaned. Encryption codes will be held securely in the MRC/Wits Agincourt Research Unit under the guardianship of the data manager. After completion of data collection, cleaning and encryption, the data files will be placed with other legacy data on the data warehouse server at the Agincourt research site.

All data sets derived from this project will be made publicly available within 1 year of the completion of data collection and cleaning. Secondary data users will submit a request for data access to the data custodians, appointed by the project principal investigators, by completing an online form. If the request falls within the bounds of appropriate data access requests as specified in the ‘MRC principles for access to, and use of, MRC funded research data’, then it will be approved. Collaboration with the original investigators in resulting publications will be encouraged.

A full analysis plan will be agreed upon with the management team and the trial steering committee before the analyses are begun. The primary analysis will be on intention to treat and will be carried out using STATA 13 software (College Station, TX, USA). Up to this point, clinic identifiers will be encrypted so that the primary analysis is carried out in blinded fashion regarding which clinics are the intervention clinics. In descriptive analyses, frequency distributions of categorical variables and summary measures of continuous variables will be reported. Baseline values in the intervention and usual care arms will be described.

To allow for confounding, the two-stage regression model will be used for analysis of the binary primary outcome ([27], pages 163--198). First, two logistic regression models for control and intervention clusters that include covariates will be fitted separately. Covariates in the two models will both be cluster-level factors (for example, clinic size) and aggregated individual-level factors (for example, sex and age). Second, observed and fitted values for each cluster will be compared by computing residuals. We expect relatively few missing values, especially for individual demographic data, because this trial is taking place in an established HDSS site with a rigorous quality assurance system.

### Objectives and approach of the process evaluation

Given the importance of context, process and actors in the performance of the health care system [28], we cannot assume that the results of a health services randomised controlled trial will be transferable to other health care settings. We are therefore undertaking an extensive process evaluation and adopting a critical realism approach in which the predominant question is, what works for whom under what conditions? This approach acknowledges that preexisting health care system structures and processes affect, and are affected by, the intervention actors. In this evaluation, we aim to understand the causal processes of change. We will take a realist approach in the evaluation and explore the ‘mechanism’ by which the intervention has its effect [29]. The following are the objectives of the process evaluation:

1. To examine how different aspects of the intervention function in the different clinics and the mechanisms by which the intervention affected or failed to affect the primary outcome
2. To examine the extent to which the lay health workers and other clinic staff were able to work within the context of the complex adapting system of a primary health care facility, by learning, interacting and self-organising to establish sustained improvements in care
3. To explain how the implementation processes shaped the intervention and its functioning
4. To explain the role of the local context in determining outcomes

### Data collection and analysis for the process evaluation

In the study, we will use a range of qualitative methods, including interviews and observation. As is standard in qualitative methods, sampling will be purposive, designed to ensure representation of a range of views and inputs. Each clinic and its attending population will be treated as a single case, and a case study approach will be used to compare and contrast experiences in the four intervention clinics. Combining qualitative and quantitative data will allow the development of within- and across-clinic analyses to explain and interpret outcomes.

Data on the clinic use of individuals will be collected through a linkage system, where consenting patients’ clinical details will be linked to the demographic surveillance system. In addition, we will collect a range of quantitative and qualitative data for the process evaluation. Quantitative data will include brief exit interviews with patients who have attended the chronic disease clinic and have a diagnosis of hypertension. We will ask whether they had their blood pressure measured, what advice they were given, whether they have been given any medication and whether a return visit has been booked. Nursing staff in the clinics will be asked to complete a structured questionnaire on their motivation.

Qualitative data will include nonparticipant observation and repeated interviews with trial employees and health service staff, including clinic supervisors and subdistrict staff. Three purposively selected groups of people in the community will be followed up with in-depth interviews. There will be three observation visits to each clinic over the period of the intervention to observe the operation of the intervention activities, to describe patient pathways and to describe the health care system facilitators and barriers to hypertension care. Throughout the period of the intervention, the lay health workers and implementation manager will be interviewed monthly to capture information on the functioning of the intervention; the usefulness of the intervention activities; adaptations to the context, barriers and facilitators to care; the relationship between the various actors; and other changes taking place in the clinic.

Three cohorts of community members will be purposively identified for two in-depth interviews at around 3 to 5 months after the initiation of the trial and again at around 12 to 15 months. The first cohort will comprise both patients who only intermittently adhere to their medication and patients who have a high level of adherence. The second cohort will comprise individuals who report that they normally attend one of the clinics in the control arm of the study and report, when interviewed, that they have hypertension. The third cohort will include individuals with raised blood pressure upon measurement who either do not report that they have hypertension or who know their diagnosis but are not taking treatment, which maybe because they are not adherent to prescribed treatment or because they have not been prescribed any treatment.

### Economic evaluation

A partial economic evaluation will be undertaken alongside the trial. As there is potential for multiple key outcomes that cannot be aggregated into a single outcome, a cost–consequence analysis will be the primary economic study undertaken to obtain an array of output measures alongside the costs. This will enable us to show the trade-offs associated with each scenario. We will collect patient-level costing data to examine the cost implication for the public sector as the funder of primary health care, as well as costs to the patient of participating in the intervention. Salary and training costs for the lay health workers and experienced nurse, as well as communication costs (such as telephone calls, SMS) will be included in the intervention cost, as well as the cost of additional services delivered by the health facility. Costs associated with the government’s ICDM programme, which is common across both intervention and comparator sites, will not be included in the analysis. Costs (in South African rand) will be collected over the course of the study, and all prices will be inflation-adjusted to the final year of the study. Nonparametric bootstrapping will be used to assess the effect of variation in patient-level outcomes on the costing results.

## Discussion

We have faced a number of challenges in designing this trial. We already knew that at least half of the people with hypertension were not aware of their condition and therefore were not receiving regular treatment [30], and we wanted to target those people as well as the people already in the clinic system. Also, until very recently, no clinic records of individual patients were kept, and the new recordkeeping system was of variable quality. It was apparent, therefore, that a simple clinic record–based measure would not be possible as the primary outcome. We therefore decided to use baseline and end-of-intervention population surveys to measure change. This created a further challenge of carrying out a large-enough survey to achieve adequate power quickly enough to obtain a realistic baseline measure.

Because the intervention involved extra resources for some clinics, we were concerned that the process of randomising the clinics could lead to suspicion about whether it was truly random and thereby create negative feelings. We therefore held a public meeting at which the randomisation took place.

To ensure that the intervention achieves the best possible outcome, the implementation manager will aim to increase clinic staff’s awareness of patient constraints, adapt activities to suit the local context and obtain local commitment. This engagement may change the local clinic context, and context and intervention are likely to coevolve. As a result, it is not realistic to attempt to maintain rigid adherence to a predetermined protocol. It is therefore important to document the evolution of the intervention, which is the reason for the extensive process evaluation. At the same time, however, to maximise the external validity of the study, it is important to keep the intervention realistically close to the type of intervention that might be feasible in a low- or middle-income country. This has led to two important dilemmas for the team. First is the extent to which findings from the process evaluation should be fed back to the implementation manager and lay health workers, as standard government policy implementation does not receive the benefit of ‘real-time’ evaluation to enable improvement. Second is the extent to which the trial team should intervene to provide extra resources where the lack of resources is preventing the effective functioning of the lay health workers. In regard to both of these dilemmas, we are regularly making day-to-day decisions, balancing the need for external validity with the practicalities of getting an intervention to run in a challenging environment. All these decisions are being carefully recorded so that we can provide a fully informed report at the end of the trial.

### Trial status

Because of the design of the trial, it is difficult to define the status of recruitment. There are eight clinics, four of which receive the intervention and four which do not. They have been randomised, and the lay health workers have been in place since February to March 2014. Although the unit of randomisation is a clinic, the participants could most appropriately be considered to be the people using the clinics. They are participating or declining to participate by consenting to their data being linked to census data and by consenting to complete an exit questionnaire or permit their consultation to be observed as they attend the clinics. In that sense, we are just over halfway through recruitment (10 months of an 18-month period). The outcome is measured by population surveys at baseline and at the end of the intervention. If the people in those surveys are considered to be the participants, then we have recruited half (one survey). A separate group of around the same number of people will be asked to complete the survey at the end. The trial status was most recently updated 6 November 2014.

## Electronic supplementary material

Additional file 1: **Questionnaire to be used in two population surveys.** (PDF 631 KB)

## Abbreviations

DBP: Diastolic blood pressure
HDSS: Health and Demographic Surveillance System
ICDM: Integrated chronic disease management
LHW: Lay health worker
MRC: Medical Research Council
SBP: Systolic blood pressure
TIA: Transient ischaemic attack.
