# Supplementary material for: The Nkateko health service trial to improve hypertension management in rural South Africa: study protocol for a randomised controlled trial
Source: Trials. 2014 Nov 7;15:435. doi: 10.1186/1745-6215-15-435 (PMC4289183; doi:10.1186/1745-6215-15-435)
Supplement: Supplementary file 1 — Additional file 1: Questionnaire to be used in two population surveys. (PDF 631 KB) [file 13063_2014_2361_MOESM1_ESM.pdf]

NKATEKO TRIAL HYPERTENSION SURVEILLANCE

Nkateko –v6
2013-09-10

Study no:	
Fieldworker:	1 1 1 1
Visit Date:	Y Y Y Y M M D D
Village:	1 1
Dwelling:	1 1 1 1 A

**0a**	Mbuyelo wa mbhurisano<br>*Interview outcome*	**1** = Interview complete<br>**2** = Interview partly completed (write down why not completed)<br>**3** = Person not found<br>**4** = Person pregnant<br>**5** = Person dead<br>**6** = Refusal (write down reason for refusal if known)<br>**7** = Sample not used/ Ineligible (write down why ineligible)	**0a**	1
**0b**	Comment			

Yana emahlweni na ntirho tani hi laha papilla ra mpfumelelo ri vuleke ha kona.
Now carry out the process of getting informed consent as instructed.

Endla leswi landzelaka, lulamisela mungheneleri ku kamberiwa ro sungula ''high blood" (a tshami eka ndhawu ya kahle na voko ra yena) loko u ri karhi u teka ''high blood" ya yena ka nharhu vutisa swi vutiso leswi laha hansi.
Next, prepare participant to have the first blood pressure measurement (sitting with arm in correct position) and while taking the three blood pressure measurements, ask the questions below.

Manghenelelo ya ndzavisiso: Swivutiso leswi landzelaka swi lava ku tiva mayelana na rihanyu na mahanyelo ya wena mayelana na mabelo ya mbilu (high blood), vuvabyi bya chukele, xikan'we na mavabyi man'wana lawa ya nga tshungulekiki. Hikwalaho ka leswi nhlayo yo tala ya vanhu va xinuna na va xisati va nghenelelaka eka xitadi lexi, swin'wana swa swivutiso swi nga ka swi nga ku khumbi. Hambi swi ri tano hi vutisa swivutiso swo fana eka vangheneleri hinkwavo. Tinhlamulo ta wena hinkwato ti ta va xihundla. Tinhlamulo ta wena ti ta tirhisiwa eka nomboro ya wena ya xihundla ya xitadi, ti nge tirhisiwi eka vito ra wena.

Introduction to the questionnaire: The following questions ask about your health and lifestyle factors associated with hypertension, diabetes and other chronic diseases. Because a broad range of men and women are participating in the study, some questions in this interview may not apply to you. However we ask the same questions to all participants. Your answers are completely confidential. Your responses are linked only to a confidential study number, not to your name.

MATIMU YA MA TLAKUKELO YA NGATI / HISTORY OF RAISED BLOOD PRESSURE

**1**	U tshama u kamberiwa mapopelo ya ngati hi dokodela, muongori kumbe unwana wa mutirhela ri hanyu?<br>*Have you ever had your blood pressure measured by a doctor, nurse or other health worker?*	**1** = Yes<br>**2** = No	**1**	1
**2**	Xana utshama u byeriwa hi dokodela, muongori kumbe unwana wa mutirhela rihanyu leswo ngati ya wena yi le henhla kumbe high blood?<br>*Have you ever been told by a doctor, nurse or other health worker that you have raised blood pressure or hypertension?*	**1** = Yes<br>**2** = No	**2**	1
**3**	xana u kumile kumbe ku nyikiwa maphilisi ya mapopelo ya ngati hi dokodela, muongori kumbe mutirhi un'wana wa swa rihanyu eka mavhiki mambirhi lawa ya hundzeke?<br>*Have you received any drugs for high blood pressure prescribed by a doctor, nurse or other health worker in the last two weeks?*	**1** = Yes<br>**2** = No➔ **Q4**	**3**	1

**3a**	(kombela ku vona phakete ya maphilisi, endla xirhendzevutana eka maphililisi lawa ku nga wona laha hansi)<br>*Yes. (ask to see packet)* **1** = Yes, seen<br>**2** = Yes, not seen, **3** = No	**1** = Hydroclorotiazide (HCTZ, RIDAC)	
		**2** = Perindopril (Prexum, Coversyl)	
		**3** = Perindopril + Indapamide (Prexum plus, Coversyl plus)	
		**4** = Enalapril (Pharmapress)	
		**5** = Atenolol (Tenblocka)	
		**6** = Nifedipine (Slow release, Adalat XL, Am loc	
		**7** = Other (write name) ➔ **Q3b**	
**3b**	If other, specify		

MATIMU YA VUVABYI BYA CHUKELA / HISTORY OF DIABETES

**4**	Xana u tshama u kamberiwa chukela ra le ngatini ya wena hi dokodela, muongori kumbe u nwana wa mutirhi wa swa rihanyu?<br>*Have you ever had your blood sugar measured by a doctor, nurse or other health workers?*	**1** = Yes<br>**2** = No	**4**	1
**5**	Xana u tshama u byeriwa hi dokodela, muongori kumbe mutirhi un'wana wa swa rihanyu leswaku chukele ra wena e ngatini ri tlakukile?<br>*Have you ever been told by a doctor, nurse or other health worker that you have raised blood sugar or diabetes?*	**1** = Yes<br>**2** = No	**5**	1

**6**	Xana u kumile maphilisi ~~kumbe~~ kumbe ku thlaviwa (jekiseni) ya mavabyi ya chukele ku suka eka dokodela, muongori kumbe mutirhi un'wana wa swa rihanyu eka mauhiki mambirhi lama hundzeke?<br>*Have you received any drugs or injections for diabetes prescribed by a doctor, nurse or other health worker in the last two weeks?*	**1** = Yes<br>**2** = No ➔ **Q7**	**6**	1

6a Ina (kombela ku vona phakete ya maphilisi kutani u sekela, endla xirhendzevutana) maphilisi lawa ku nga wona laha hansi)
Yes, (ask to see packet) **1** = Yes, seen **2** = Yes, not seen **3** = No

**1** = Glibenclamide (Glycon, Daonil)	
**2** = Gliclazide (Diamicron)	
**3** = Metformin (Glicophage)	
**4** = Other (write name) ➔ **Q6b**	

6b If other, specify

MATIMU YA XITOROKU / HISTORY OF STROKE

**7**	Xana u tshama u byeriwa hi dokodela, muongori kumbe mutirhi wa swa rihanyu leswaku una xitoroku?<br>*Have you ever been told by a doctor, nurse or other health worker that you have had a stroke?*	**1** = Yes<br>**2** = No	**7**	1
**8**	Xana u tshama u nyikiwa murhi wo tshungula xitoroku?<br>*Did you ever receive medical treatment for stroke?*	**1** = Yes<br>**2** = No ➔ **Q10**	**8**	1

9a Xana u nga va u kume vutshunguri bya wena eka mavabyi ya ku oma swirho (xitoroku) eka tindhawu leti landzelaka? (rhendzela leswi hlamuriweke/hlawula)
Where did you get treatment for your stroke from any of the following? **1** = Yes **2** = No

**1** = Public clinic /Eka kliniki ya mfumo	
**2** = Hospital / Exibedhlela	
**3** = Private doctor or nurse / Eka dokodela kumbe muongori loyi a nga ri ki wa mfumo	
**4** = Traditional healer/ Eka tinyanga (dokodela wa xintu)	
**5** = Other (write name)/ kun'wana (tsala vito) ➔ **Q9b**	

9b If other, specify

MATIMU YA TSANDZEKA KU POMPA KAHLE NGATI / HISTORY OF HEART FAILURE

**10**	Xana u tshama u byeriwa hi dokodela, muongori kumbe mutirhi un'wana wa swa rihanyu leswaku mbilu ya wena ya tsandzeka ku pompa ngati hi ndlela ya kahle minkarhi yin'wana?<br>*Have you ever been told by a doctor, nurse or other health worker that you have heart failure?*	**1** = Yes<br>**2** = No	**10**	1
**11**	Xana u kumile vutshunguri eka vuvabyi bya wena bya mbilu loko yi tsandzeka ku pompa kahle ngati?<br>*Have you ever received medical treatment for heart failure?*	**1** = Yes<br>**2** = No ➔ **Q13**	**11**	1

12a Xana vutshunguri eka mavabyi ya wena ya ku tsandzeka ka mbilu ya wena ku pompa ngati u byi kume kwihi? (rhendzela nhlamulo leyi nga yona)
Where did you get treatment for your heart failure from any of the following? **1** = Yes **2** = No

**1** = Public clinic /Eka kliniki ya mfumo	
**2** = Hospital / Exibedhlela	
**3** = Private doctor or nurse / Eka dokodela kumbe muongori loyi a nga ri ki wa mfumo	
**4** = Traditional healer/ Eka tinyanga (dokodela wa xintu)	
**5** = Other (write name)/ kun'wana (tsala vito) ➔ **Q12b**	

12b If other, specify

MATIMU YA ANGINA / HISTORY OF ANGINA

**13**	Xana u tshama u byeriwa hi dokodela kumbe muongori leswaku u na angina?<br>*Have you ever been told by a doctor or nurse that you have angina or angina pectoris?*	**1** = Yes<br>**2** = No	**13**	1
**14**	Xana u tshama u kuma vu tshunguri ka vuvabyi bya angina?<br>*Have you ever received medical treatment for angina?*	**1** = Yes<br>**2** = No➔ **Q16**	**14**	1

15a Xana vutshunguri bya mavabyi ya angina byi kumile kwihi? (hlawula ka leswi landzelaka)
Where did you get treatment for your angina from any of the following **1** = Yes **2** = No

**1** = Public clinic /Eka kliniki ya mfumo	
**2** = Hospital / Exibedhlela	
**3** = Private doctor or nurse / Eka dokodela kumbe muongori loyi a nga ri ki wa mfumo	
**4** = Traditional healer/ Eka tinyanga (dokodela wa xintu)	
**5** = Other (write name)/ kun'wana (tsala vito) ➔ **Q15b**	

15b If other, specify

MATIMU YA HEART ATTACK/ HISTORY OF HEART ATTACK

**16**	Xana u tshama u byeriwa hi dokodela, muongori kumbe mutirhi un'wana wa swa rihanyu leswaku u ni heart attack?<br>*Have you ever been told by a doctor or a nurse or a health care worker that you have had a heart attack?*	**1** = Yes<br>**2** = No	**16**	1
**17**	Xana u kumile vutshunguri endzhaku ka loko u khomiwile hi heart attack?<br>*Did you receive medical treatment for your heart attack?*	**1** = Yes<br>**2** = No ➔ **Q19**	**17**	1
**18a**	Xana vutshunguri u byi kumile ekati ndawu leti ladzelaka? (rhendzela nhlamulo leyi ku nga yona)?<br>*Where did you get treatment for your heart attack from any of the following*<br>**1** = Yes **2** = No	**1** = Public clinic /Eka kliniki ya mfumo<br>**2** = Hospital / Exibedhlela<br>**3** = Private doctor or nurse / Eka dokodela kumbe muongori loyi a nga ri ki wa mfumo<br>**4** = Traditional healer/ Eka tinyanga (dokodela wa xintu)<br>**5** = Other (write name)/ kun'wana (tsala vito) ➔ **Q18b**		
**18b**	If other, specify			
**19**	Xana eka vatswari va wena kumbe vamakwenu va ngati, a ngava ari kona a nga tshama a kumeka a ri na xiphiqo xa mbilu kumbe xitiroku (tatana, manana, buti kumbe sesi)?<br>*Has any of your first degree family members (father, mother, brothers and sisters) been diagnosed as having a heart problem or stroke?*	**1** = Yes<br>**2** = No ➔ **Q20**	**19**	1
**19b**	Xana a ku ri tatana kumbe buti loko va ha ri hansi ka malembe ya 55?<br>*Was it your father or brother at an age younger than 55 years?*	**1** = Yes<br>**2** = No	**19b**	1
**19c**	Xana a ku ri manana kumbe sesi loko va ha ri hansi ka malembe ya 65?<br>*Was it your mother or sister at an age younger than 65 years?*	**1** = Yes<br>**2** = No	**19c**	1
**20**	Xana u tshame u dzaha fole ra sigarete?<br>*Have you ever smoked any cigarettes?*	**1** = Yes<br>**2** = No ➔ **Q23**	**20**	1
**21**	Xana eka nkarhi wa sweswi wa ha dzaha (kumbe minkarhi yin'wana) fole ra tisigarete?<br>*Do you currently smoke (you smoke if you have the chance) any cigarettes?*	**1** = Yes<br>**2** = No ➔ **Q23**	**21**	1
**22**	Eka masiku ya makume-manharhu (30), xana u dzahe kangani?<br>*During the past 30 days, how frequently have you smoked any cigarettes?*	**1** = Daily<br>**2** = 5-6 days per week<br>**3** =1-4 days per week<br>**4** =1-3 days per month	**22**	1
**23**	Xana u tshame u endzela kliniki ya mfumo?<br>*Have you ever attended public clinic?*	**1** = Yes<br>**2** = No ➔ **Q26**	**23**	1
**24**	Loko u ta ya ra mahetelelo ekliniki, xana a wu ye ka yihi kliniki (rhendzela yin'we ntsena)<br>*When you last attended a clinic, which clinic did you attend?*	**1**= Agincourt<br>**2** = Arthurstone<br>**3** = Belfast<br>**4** = Bhubezi<br>**5** = Cork<br>**6** = Cunningmore<br>**7** = Kildare<br>**8** = Lillydale<br>**9** = Justicia<br>**10** = Oakley<br>**11** = Thulamahashe<br>**12** = Xanthia<br>**13** = Other (write name) ➔ **Q24a**	**24**	1 1
**24a**	If other, specify			

**25**	Xana hi yihi kliniki leyi u yaka eka yona hi xitalo loko u tshuka u lava ku ya kliniki? (rhendzela yin'we ntsena)<br><br>*Which clinic do you USUALLY attend when you need to go to a clinic?*	**1**= Agincourt<br>**2** = Arthurstone<br>**3** = Belfast<br>**4** = Bhubezi<br>**5** = Cork<br>**6** = Cunningmore<br>**7** = Kildare<br>**8** = Lillydale<br>**9** = Justicia<br>**10** = Oakley<br>**11** = Thulamahashe<br>**12** = Xanthia<br>**13** = Other (write name) ➔ **Q25a**	**25**
**25a**	If other, specify		
**26**	Eka tin'wheti leti landzelaka hi ta tsakela ku burisana na vanhu van'wana mayelana na rihanyu ra vona. Xana u nga swi tsakela leswaku hi thlela hi ku endzela na kambe ku ta burisana na wena?<br>*We would like to talk to a few people about their health again in a few months' time. Would you allow us to come to talk to you again?*	**1** = Yes, you may come to talk to me again ➔ **Q26a**<br>**2** = No, do not come to talk to me again	**26**
**26a**	If yes, ask for respondent phone number		
	**BLOOD PRESSURE & PULSE**		
**27**	Reading 1: Systolic (SYS)	**(Answer in mm Hg)**	**27a**
	Reading 1: Diastolic (DIA)	**(Answer in mm Hg)**	**27b**
	Reading 1: Heart Rate/ Pulse	**(Beats per minute)**	**27c**
**28**	Reading 2: Systolic (SYS)	**(Answer in mm Hg)**	**28a**
	Reading 2: Diastolic (DIA)	**(Answer in mm Hg)**	**28b**
	Reading 2: Heart Rate/ Pulse	**(Beats per minute)**	**28c**
**29**	Reading 3: Systolic (SYS)	**(Answer in mm Hg)**	**29a**
	Reading 3: Diastolic (DIA)	**(Answer in mm Hg)**	**29b**
	Reading 3: Pulse	**(Beats per minute)**	**29c**
	**U nga si teka ngati eka rintiho/mpimanyeto wa thonsi ra ngati.**<br>***Before taking the finger prick/ blood drop measurements***		
**30**	Xana u nwile maphilisi ya insulin kumbe man'wana (mirhi) leyi u nyikiweke hi dokodela, muongori kumbe mutirhi un'wana wa swa rihanyu ya chukele ra wena leri tlakuke namunthla?<br>*Today, have you taken insulin or other drugs (medication) that have been prescribed by a doctor, a nurse or other health worker for raised blood glucose?*	**1**= Yes<br>**2** = No	**30**
**31**	Xana u nga va u nwile yin'wana mirhi eka tiawara ta khume-mbirhi leti hundzeke?<br>*Have you taken any other medication in the last 12 hours?*	**1** = Yes<br>**2** = No ➔ **Q33**	**31**
**32**	Xana a ku ri mirhi kumbe maphilisi ya njani?<br>*What is this medication?*	**32**	
**33**	Eka tiawara ta khume-mbirhi leti hundzeke, xana u nga va u dyile kumbe ku nwa swo karhi?<br>*During the past 12 hours, have you had anything to eat or to drink?*	**1**= Yes<br>**2** = No	**33**
	**BLOOD LIPIDS / MAFURHA YA LE NGATINI**		
**34**	Total Cholesterol (mmol/L)		**34**
	**BLOOD GLUCOSE / CHUKELA RA LE NGATINI**		
**35**	Glucose in blood (mmol/L)		**35**
	**WAIST**		
**36**	Waist circumference	**(Answer in centimeters (cm))**	**36**
